# Supplementary material for: Incidence of Mortality, Acute Kidney Injury and Graft Loss in Adult Kidney Transplant Recipients with Coronavirus Disease 2019: Systematic Review and Meta-Analysis
Source: J Clin Med. 2021 Nov 4;10(21):5162. doi: 10.3390/jcm10215162 (PMC8584628; doi:10.3390/jcm10215162)

Supplemental Files

Supplemental Table Legends

Supplemental Table S1. PRISMA checklist

Supplemental Table S2. Details of Search Strategy results for each database

Supplemental Table S3. Primary reasons for exclusion of excluded studies

Supplemental Table S4. Newcastle-Ottawa Scale assessment of the included studies

Supplemental Figure Legends

Supplemental Figure S1. PRISMA Flow Diagram

Supplemental Figure S2. Funnel plot for publication bias assessment

Supplemental Table S1 PRISMA checklist

Section and Topic	Item #	Checklist item	Location where item is reported
**TITLE**			
Title	1	Identify the report as a systematic review.	Page 1
**ABSTRACT**			
Abstract	2	See the PRISMA 2020 for Abstracts checklist.	Page 2
**INTRODUCTION**			
Rationale	3	Describe the rationale for the review in the context of existing knowledge.	Page 3
Objectives	4	Provide an explicit statement of the objective(s) or question(s) the review addresses.	Page 3
**METHODS**			
Eligibility criteria	5	Specify the inclusion and exclusion criteria for the review and how studies were grouped for the syntheses.	Page 4
Information sources	6	Specify all databases, registers, websites, organisations, reference lists and other sources searched or consulted to identify studies. Specify the date when each source was last searched or consulted.	Page 4
Search strategy	7	Present the full search strategies for all databases, registers and websites, including any filters and limits used.	Page 4
Selection process	8	Specify the methods used to decide whether a study met the inclusion criteria of the review, including how many reviewers screened each record and each report retrieved, whether they worked independently, and if applicable, details of automation tools used in the process.	Page 4
Data collection process	9	Specify the methods used to collect data from reports, including how many reviewers collected data from each report, whether they worked independently, any processes for obtaining or confirming data from study	Page 4-5

Section and Topic	Item #	Checklist item	Location where item is reported
		investigators, and if applicable, details of automation tools used in the process.	
Data items	10a	List and define all outcomes for which data were sought. Specify whether all results that were compatible with each outcome domain in each study were sought (e.g. for all measures, time points, analyses), and if not, the methods used to decide which results to collect.	Page 5
	10b	List and define all other variables for which data were sought (e.g. participant and intervention characteristics, funding sources). Describe any assumptions made about any missing or unclear information.	Page 5
Study risk of bias assessment	11	Specify the methods used to assess risk of bias in the included studies, including details of the tool(s) used, how many reviewers assessed each study and whether they worked independently, and if applicable, details of automation tools used in the process.	Page 5-6
Effect measures	12	Specify for each outcome the effect measure(s) (e.g. risk ratio, mean difference) used in the synthesis or presentation of results.	Page 5
Synthesis methods	13a	Describe the processes used to decide which studies were eligible for each synthesis (e.g. tabulating the study intervention characteristics and comparing against the planned groups for each synthesis (item #5)).	Page 5
	13b	Describe any methods required to prepare the data for presentation or synthesis, such as handling of missing summary statistics, or data conversions.	Page 5
	13c	Describe any methods used to tabulate or visually display results of individual studies and syntheses.	Page 5
	13d	Describe any methods used to synthesize results and provide a rationale for the choice(s). If meta-analysis was performed, describe the model(s), method(s) to identify the presence and extent of statistical heterogeneity, and	Page 5

Section and Topic	Item #	Checklist item	Location where item is reported
		software package(s) used.	
	13e	Describe any methods used to explore possible causes of heterogeneity among study results (e.g. subgroup analysis, meta-regression).	Page 5
	13f	Describe any sensitivity analyses conducted to assess robustness of the synthesized results.	Page 5
Reporting bias assessment	14	Describe any methods used to assess risk of bias due to missing results in a synthesis (arising from reporting biases).	Page 5
Certainty assessment	15	Describe any methods used to assess certainty (or confidence) in the body of evidence for an outcome.	N/A
**RESULTS**			
Study selection	16a	Describe the results of the search and selection process, from the number of records identified in the search to the number of studies included in the review, ideally using a flow diagram.	Page 6
	16b	Cite studies that might appear to meet the inclusion criteria, but which were excluded, and explain why they were excluded.	Page 6
Study characteristics	17	Cite each included study and present its characteristics.	Page 6
Risk of bias in studies	18	Present assessments of risk of bias for each included study.	Page 7
Results of	19	For all outcomes, present, for each study: (a) summary statistics for each group (where appropriate) and (b) an	Page 6-7

Section and Topic	Item #	Checklist item	Location where item is reported
individual studies		effect estimate and its precision (e.g. confidence/credible interval), ideally using structured tables or plots.	
Results of syntheses	20a	For each synthesis, briefly summarise the characteristics and risk of bias among contributing studies.	Page 6-7
	20b	Present results of all statistical syntheses conducted. If meta-analysis was done, present for each the summary estimate and its precision (e.g. confidence/credible interval) and measures of statistical heterogeneity. If comparing groups, describe the direction of the effect.	Page 6-7
	20c	Present results of all investigations of possible causes of heterogeneity among study results.	Page 6-7
	20d	Present results of all sensitivity analyses conducted to assess the robustness of the synthesized results.	Page 6-7
Reporting biases	21	Present assessments of risk of bias due to missing results (arising from reporting biases) for each synthesis assessed.	Page 7
Certainty of evidence	22	Present assessments of certainty (or confidence) in the body of evidence for each outcome assessed.	N/A
**DISCUSSION**			
Discussion	23a	Provide a general interpretation of the results in the context of other evidence.	Page 8
	23b	Discuss any limitations of the evidence included in the review.	Page 9-10
	23c	Discuss any limitations of the review processes used.	Page 9-10
	23d	Discuss implications of the results for practice, policy, and future research.	Page 9-10
**OTHER INFORMATION**			

Section and Topic	Item #	Checklist item	Location where item is reported
Registration and protocol	24a	Provide registration information for the review, including register name and registration number, or state that the review was not registered.	Page 4
	24b	Indicate where the review protocol can be accessed, or state that a protocol was not prepared.	Page 4
	24c	Describe and explain any amendments to information provided at registration or in the protocol.	Page 4
Support	25	Describe sources of financial or non-financial support for the review, and the role of the funders or sponsors in the review.	Page 11
Competing interests	26	Declare any competing interests of review authors.	Page 11
Availability of data, code and other materials	27	Report which of the following are publicly available and where they can be found: template data collection forms; data extracted from included studies; data used for all analyses; analytic code; any other materials used in the review.	N/A

From: Page MJ, McKenzie JE, Bossuyt PM, Boutron I, Hoffmann TC, Mulrow CD, et al. The PRISMA 2020 statement: an updated guideline for reporting systematic reviews. BMJ 2021;372:n71. doi: 10.1136/bmj.n71

Supplemental Table S2. Details of Search Strategy results for each database

**Pubmed through June 08st, 2021**	
#1	(((Kidney Transplantation) OR (Kidney transplant)) OR (Renal transplantation)) OR (Renal transplant)
#2	((COVID-19) OR (SARS-CoV-2)) OR (coronavirus)
#3	#1 AND #2
#4	#3 + Filters: Humans, Adult: 19+ years, English, from 2019 - 2021 , Result: 371
**EMbase through June 08st, 2021**	
#1	'covid 19'/exp OR 'covid 19'
#2	'sars cov 2'
#3	('kidney'/exp OR kidney) AND ('transplantation'/exp OR transplantation)
#4	#1 AND #2
#5	#3 AND #4, Result: 496
**Medline through June 08st, 2021**	
#1	kidney transplantation.mp. or exp Kidney Transplantation /
#2	renal transplantation.mp. or exp Kidney Transplantation /
#3	1# or 2#
#4	COVID-19.mp. or exp COVID-19 /
#5	3# and 4# , Result: 385

Supplemental Table S3. Primary reasons for exclusion of excluded studies

No	First Author	Journal	Year	Title	Primary Reason for Exclusion	Include
1	Andrea Angeletti	Clinical Transplantation	2020	Risk of COVID-19 in young kidney transplant recipients. Results from a single-center observational study	Not adult population	N
2	Fabio Vistoli	Journal of Nephrology	2020	COVID-19 and kidney transplantation: an Italian Survey and Consensus	Insufficient information	N
3	Filipa Silva	Transpl Infect Dis.	2021	SARS-CoV-2 infection in kidney transplant recipients: Early report of five cases	Different outcome of interest	N
4	Hui Zhang	EUROPEAN UROLOGY	2020	Identification of Kidney Transplant Recipients with Coronavirus Disease 2019	Insufficient information	N
5	Ilies Benotmane	Am J Transplant	2020	In-depth virological assessment of kidney transplant recipients with COVID-19	Duplication cohort	N
6	Julio Pascual	EUROPEAN UROLOGY	2020	COVID-19–related Mortality During the First 60 Days After Kidney Transplantation	Duplication cohort	N
7	Laila A. Viana	Transplantation	2021	Kidney Transplantation in Patients With SARS-CoV-2 Infection: A Case Series Report	Kidney Transplantation after infection	N
8	Marcus R. Pereira	Am J Transplant	2020	COVID-19 in solid organ transplant recipients: Initial report from the US epicenter	Solid organ transplantation other than kidney transplant	N
9	Marie Elldin	Transplantation	2021	Initial Report From a Swedish High-volume Transplant Center After the First Wave of the COVID-19 Pandemic	Solid organ transplantation other than kidney transplant	N
10	MaríaJ.Pérez-Sáez	Am J Transplant	2020	Use of tocilizumab in kidney transplant recipients with COVID-19	Duplication cohort	N
11	Marta Crespo	Am J Transplant	2020	COVID-19 in elderly kidney transplant recipients	Duplication cohort	N
12	Marta Crespo	Transplantation	2020	Respiratory and Gastrointestinal COVID-19 Phenotypes in Kidney Transplant Recipients	Duplication cohort	N
13	Maryam Ghaffari Rahbar	Transpl Infect Dis.	2021	Low rate of COVID-19 pneumonia in kidney transplant recipients—A battle between infection and immune response?	Included not COVID-19 confirm cases	N

14	Olivier Thaunat	Kidney International	2020	IMPact of the COVID-19 epidemic on the moRTAlity of kidney transplant recipients and candidates in a French Nationwide registry sTudy (IMPORTANT)	Insufficient information (No actual infected number)	N
15	Rommel Ravanan	Am J Transplant	2020	SARS-CoV-2 infection and early mortality of waitlisted and solid organ transplant recipients in England: A national cohort study	Solid organ transplantation other than kidney transplant	N
16	Torres Ortiz	CJASN	2020	COVID-19 in Kidney Transplant Recipients: Experience from a Large Health System in Louisiana	Insufficient information (conference abstract)	N
17	S. Ali Husain	CJASN	2020	Early Outcomes of Outpatient Management of Kidney Transplant Recipients with Coronavirus Disease 2019	Different outcome of interest	N
18	Stephanie G. Yi	Transplantation	2020	Early Experience With COVID-19 and Solid Organ Transplantation at a US High-volume Transplant Center	Solid organ transplantation other than kidney transplant	N
19	Syed Naeem	Transpl Infect Dis.	2021	Successful recovery from COVID-19 in three kidney transplant recipients who received convalescent plasma therapy	Different outcome of interest	N

Supplemental table S4. Newcastle-Ottawa Scale assessment of the included studies

Study	Representativeness of the exposed cohort (1)	Selection of the non exposed cohort (1)	Ascertainment of exposure (1)	Demonstration that outcome of interest was not present at start of study (1)	Comparability of cohorts on the basis of the design or analysis (2)	Assessment of outcome (1)	Was follow-up long enough for outcomes to occur (1)	Adequacy of follow up of cohorts (1)	NOS_score
Abolghasemi	1	0	1	1	0	1	1	1	6
Abrishami	0	0	1	1	0	1	1	1	5
Akalin	0	0	1	1	0	1	1	1	5
Alberici	0	0	1	1	0	1	1	1	5
Azzi	0	0	1	1	0	1	1	1	5
Banerjee	1	0	1	1	0	1	1	1	6
Bell	1	1	1	1	0	1	1	1	7
Benotmane	0	0	1	1	0	1	1	1	5
Bossini	1	0	1	1	0	1	1	1	6
Caillard	1	0	1	1	0	1	1	1	6
Chavarot	1	1	1	1	0	1	1	1	7
Chen	0	0	1	1	0	1	1	1	5
Coll	1	0	1	1	0	1	1	1	6
Craig-Schapiro	0	1	1	1	0	1	1	1	6
Cravedi	1	0	1	1	0	1	1	1	6

Cristelli	0	0	1	1	0	1	1	1	5
Demir	1	0	1	1	0	1	1	1	6
Devresse	0	0	1	1	0	1	1	1	5
Dheir	0	0	1	1	0	1	1	1	5
Elec	0	0	1	1	0	1	1	1	5
Elhadedy	1	0	1	1	0	1	1	1	6
Elias	1	1	1	1	0	1	1	1	7
Favà	1	0	1	1	0	1	1	1	6
Fernández-Ruiz	0	0	1	1	0	1	1	1	5
Georgery	0	0	1	1	0	1	1	1	5
Gupta	0	0	1	1	0	1	1	1	5
Hardesty	0	1	1	1	0	1	1	1	6
Hilbrands	1	1	1	1	0	1	1	1	7
Husain	0	0	1	1	0	1	1	1	5
Jager	1	1	1	1	0	1	1	1	7
Katz-Greenberg	0	0	1	1	0	1	1	1	5
Kumaresan	0	0	1	1	0	1	1	1	5
Kute	1	0	1	1	0	1	1	1	6

Lubetzky	0	0	1	1	0	1	1	1	5
Lum	0	0	1	1	0	1	1	1	5
Mamode	1	1	1	1	0	1	1	1	7
Maritati	0	0	1	1	0	1	1	1	5
Meester	1	1	1	1	0	1	1	1	7
Mella	0	0	1	1	0	1	1	1	5
Meziyerh	0	0	1	1	0	1	1	1	5
Mohamed	0	1	1	1	0	1	1	1	6
Molaei	0	0	1	1	0	1	1	1	5
Monfared	0	0	1	1	0	1	1	1	5
Montagud-Marrahi	0	0	1	1	0	1	1	1	5
Nair	0	0	1	1	0	1	1	1	5
Oto	1	0	1	1	0	1	1	1	6
Ozturk	1	1	1	1	0	1	1	1	7
Phanish	1	1	1	1	0	1	1	1	7
Pierrotti	0	0	1	1	0	1	1	1	5
Rodriguez-Cubillo	0	0	1	1	0	1	1	1	5
Sandes-Freitas	1	0	1	1	0	1	1	1	6

Santeusanio	0	0	1	1	0	1	1	1	5
Shrivastava	0	0	1	1	0	1	1	1	5
Tejada	0	0	1	1	0	1	1	1	5
Trujillo	0	0	1	1	0	1	1	1	5
Villa	0	1	1	1	0	1	1	1	6
Villanego	1	0	1	1	0	1	1	1	6
Willicombe	0	0	1	1	0	1	1	1	5
Zhu	1	0	1	1	0	1	1	1	6

Supplemental Figure S1. PRISMA Flow Diagram

PRISMA 2009 Flow Diagram

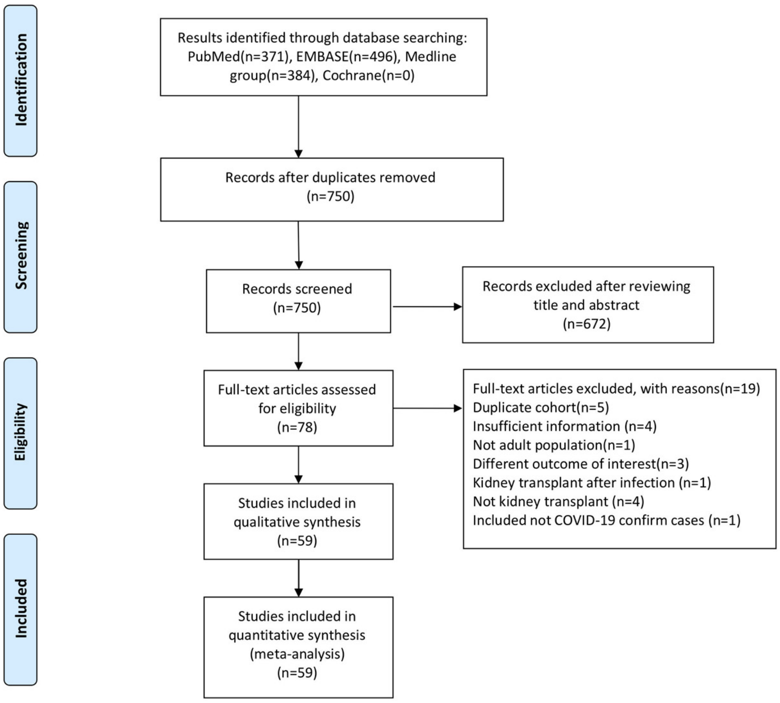

From: Moher D, Liberati A, Tetzlaff J, Altman DG, The PRISMA Group (2009). *P*referred *R*eporting *I*tems for *S*ystematic Reviews and *M*eta-*A*nalyses: The PRISMA Statement. PLoS Med 6(7): e1000097. doi:10.1371/journal.pmed1000097

For more information, visit www.prisma-statement.org.

Supplemental Figure S2. Funnel plot for publication bias assessment

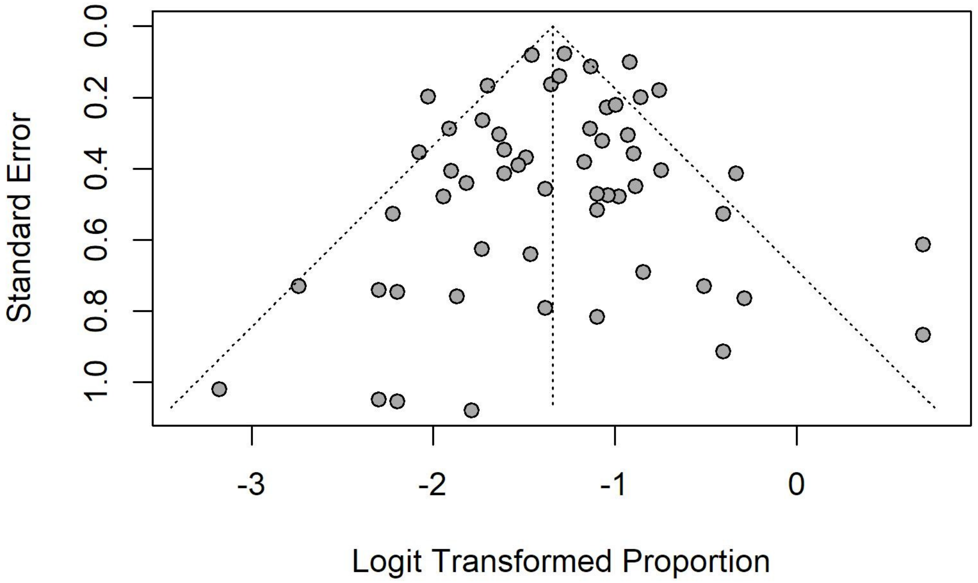

Supplement: Supplementary file 1 [file jcm-10-05162-s001.zip › jcm-1384349-supplementary.pdf]
